# Serum Homocysteine Levels and Their Relationship With Serum Vitamin B12, Folate, and Ferritin Levels in Transfusion-Dependent Thalassemic Children

**DOI:** 10.7759/cureus.69675

**Published:** 2024-09-18

**Authors:** Likhitha S, Suman Kumari, Manoj Kumar, Vikash Katewa, Pramod Sharma, Sangeeta Yadav

**Affiliations:** 1 Pediatrics, Dr. S. N. Medical College, Jodhpur, IND; 2 Obstetrics and Gynaecology, Gattani Hospital, Jodhpur, IND

**Keywords:** serum ferritin, serum folic acid, serum homocysteine, serum vitamin b12, transfusion dependent thalassemic children

## Abstract

Purpose: The purpose of this study was to assess serum homocysteine levels and their relationship with serum vitamin B12, folate, and ferritin levels in transfusion-dependent thalassemic children. This study was proposed due to a paucity of literature regarding the status of homocysteine levels in thalassemic children and their relationship with the levels of vitamins and iron overload (serum ferritin values).

Methodology: A descriptive observational study was conducted on transfusion-dependent thalassemic children aged 1-18 years, who were registered at the Thalassemia Day Care Centre (TDCC), Umaid Hospital, Dr. SN Medical College, Jodhpur, over a period of six months.

Results: A total of 100 children were enrolled in the study, with a mean age of 8.89±4.50 years. The mean pre-transfusion hemoglobin level in the last six months was 8.23±1.02 gm/dL. The mean serum levels of homocysteine, vitamin B12, folic acid, and ferritin were 10.93±3.72 µmol/L, 164.03±80.54 pg/mL, 7.69±5.77 ng/mL, and 2175.78±1341.39 ng/mL, respectively. A statistically significant negative correlation was detected between serum vitamin B12 concentration and serum homocysteine concentration (r=-0.285, p=0.004). Statistically non-significant positive correlations were detected between serum folic acid and serum homocysteine levels (r=0.033, p=0.748) and between serum ferritin and serum homocysteine levels (r=0.179, p=0.075).

Conclusion: A statistically significant negative correlation between serum homocysteine and vitamin B12 levels was noted, whereas statistically non-significant positive correlations were observed between serum homocysteine and serum folic acid levels and between serum homocysteine and serum ferritin levels.

## Introduction

Thalassemia is a genetic disorder of globin chain production that results in either a partial or complete reduction in the production of the globin chain in the hemoglobin molecule. The recommended treatment for beta-thalassemia major involves regular blood transfusions throughout life given at intervals of three to four weeks to maintain the pre-transfusion level of hemoglobin between 9 and 10.5 g/dL [1,2]. Transfusion-dependent thalassemic children are those who have received 10 or more transfusions in their lifetime.

Folate and vitamin B12 are required for normal erythropoiesis. Previous studies have shown a reduction in folate levels in thalassemic patients. Homocysteine is a surrogate marker of either vitamin B12 deficiency or folate deficiency, whereas methylmalonic acid is a surrogate marker for the detection of vitamin B12 deficiency.

Oxidative stress, defined as disruption of the equilibrium between the pro- and antioxidant systems, causes alterations in the activity of the enzymes involved in HCY metabolism [3,4]. Thalassemia is also associated with increased oxidative stress; the increase in serum ferritin due to continuous blood transfusions promotes peroxidative damage in thalassemic patients [5]. One study reported that increased oxidative stress in thalassemic children stimulates the activity of cystathionine beta synthase, which leads to increased homocysteine catabolism and reduced levels of homocysteine in thalassemic patients [6].

Since the possible alterations in plasma homocysteine levels may have serious potential consequences and there is a paucity of literature regarding the status of homocysteine levels in thalassemic children and their relationship with other vitamins, this study was planned to investigate the levels of homocysteine and their relationship with the levels of vitamin B12, folate, and ferritin in β-thalassemic patients.

## Materials and methods

This descriptive observational study was conducted from June to November 2022 at a tertiary care teaching hospital in western Rajasthan. One hundred transfusion-dependent thalassemic children aged 1-18 years, who were registered at the Thalassemic Day Care Centre (TDCC), were enrolled in the study. Children on anticonvulsants such as phenytoin, carbamazepine, and other medications such as antifolates and theophylline, and those with associated illnesses such as diabetes mellitus, thyroid dysfunction, or cancer were excluded from the study. Written informed consent was obtained from the parents/guardians of all the subjects before recruitment.

A complete detailed history, e.g., age at diagnosis of thalassemia, average number of blood transfusions received in a year, family history of thalassemia, pre-transfusion hemoglobin level, and chelation details, was collected and recorded in a predesigned proforma. Clinical features suggestive of vitamin B12 and folic acid deficiency, such as paresthesia, gait ataxia, peripheral neuropathy, hand tremors, glossitis, cheilitis, stomatitis, bald tongue, hyperpigmentation of the skin, and anorexia, were also recorded.

Blood samples were collected for the estimation of serum homocysteine, vitamin B12, folic acid, and ferritin levels. A complete blood count with peripheral blood smear details (to look for the morphology of cells such as anisocytosis and poikilocytosis, macro-ovalocytosis, hypersegmented neutrophils, leukopenia, and thrombocytopenia) was performed for each patient. Serum vitamin B12, folic acid, and ferritin levels were measured by Bechman and Coulter Access-2 Chemiluminescence Automatic Analyzer (Beckman Coulter, Inc., Brea, USA). Serum homocysteine was measured via the chemiluminescence method by ADVIA Centaur XP. The normal reference ranges of homocysteine, vitamin B12, folic acid, and ferritin were 5-15 µmol/L, 279-996 pg/mL, 5-20 ng/mL, and 29-248 ng/mL, respectively.

Continuous data were analyzed and expressed as means and standard deviations. The associations between categorical variables were tested via the chi-square test or Fisher’s exact test. Student’s t-test was also used where appropriate. Pearson’s correlation was used to study the relationships between serum homocysteine and serum vitamin B12, serum folic acid, and serum ferritin. p<0.05 was considered significant. The data were analyzed via Epi Info statistical software.

## Results

One hundred transfusion-dependent thalassemic children aged 1-18 years were enrolled in the study. The baseline characteristics of the children are presented in Table 1. The mean age of the children was 8.89±4.50 years. Of 100 children, 62 (62%) were male and 38 (38%) were female. The mean age at thalassemia diagnosis was 9.22±3.71 months. The mean average number of transfusions in the previous year was 25.88±5.25. The mean pre-transfusion Hb level in the past six months was 8.23±1.02 gm/dL. The mean serum levels of homocysteine, vitamin B12, folic acid, and ferritin were 10.93±3.72 µmol/L, 164.03±80.54 pg/mL, 7.69±5.77 ng/mL, and 2175.78±1341.39 ng/mL, respectively.

**Table 1:** Baseline characteristics of the thalassemic children (n=100).

Characteristics			Number (%)	Mean±SD
Age		1-5 years	23 (23%)	8.89±4.50 years
		5-10 years	40 (40%)	
		11-15 years	29 (29%)	
		15-18 years	8 (8%)	
Gender		Male	62 (62%)	-
		Female	38 (38%)	
Religion		Hindu	86 (86%)	-
		Muslim	14 (14%)	
History of thalassemia in siblings		Yes	14 (14%)	-
		No	86 (86%)	
Age at diagnosis of thalassemia		0-6 months	39 (39%)	9.22±4.50months
		6-12 months	44 (44%)	
		12-18 months	17 (17%)	
Average number of transfusion in a year		12-20	24 (24%)	25.88±5.25
		>20	76 (76%)	
Average pre-transfusion hemoglobin in last six months(gm/dL)		<7	7 (7%)	8.23±1.02 gm/dL
		7-10	89 (89%)	
		>10	4 (4%)	
Biochemical parameters	Serum homocysteine (µmol/L)	<5	1 (1%)	10.93±3.72 µmol/L
		5-15	89 (89%)	
		>15	10 (10%)	
	Serum vitamin B12 (pg/mL)	<279	92 (92%	164.03±80.54 pg/mL
		279-996	8 (8%)	
	Serum folic acid (ng/mL)	<5	41 (41%)	7.69±5.77 ng/mL
		5-20	53 (53%)	
		>20	6 (6%)	
	Serum ferritin (ng/mL)	29-248	3 (3%)	2175.78±1341.39 ng/mL
		>248	97 (97%)	

**Figure 1:**
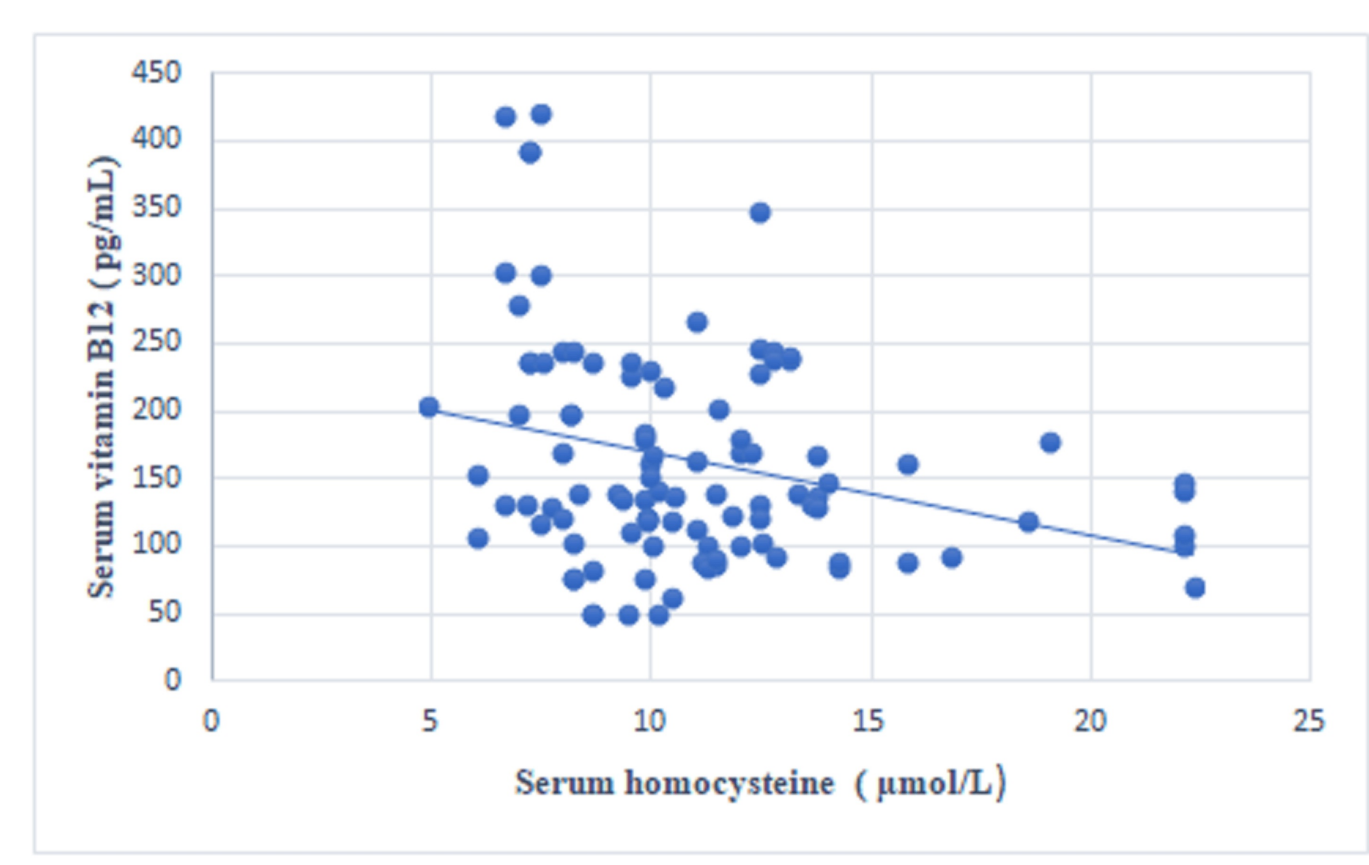
Scatter plot showing the correlation between serum homocysteine and serum vitamin B12.

**Figure 2:**
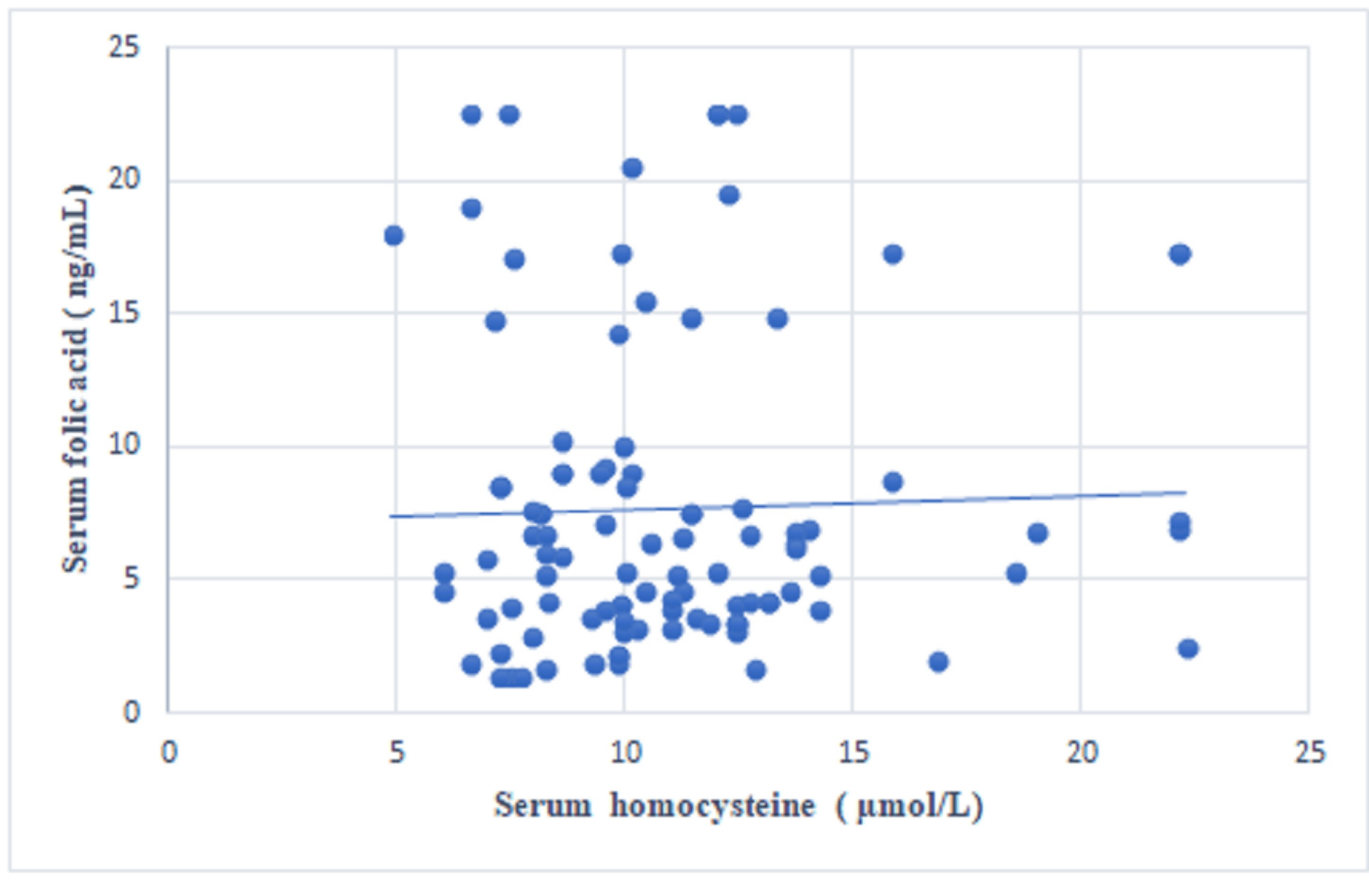
Scatter plot showing the correlation between serum homocysteine and serum folic acid levels.

**Figure 3:**
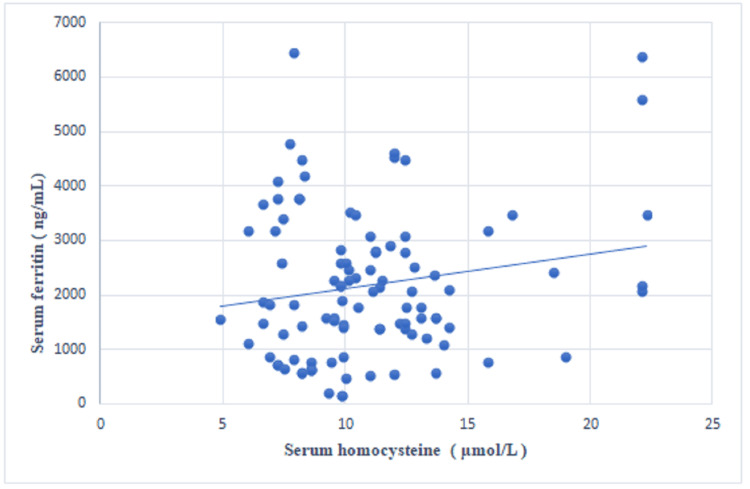
Scatter plot showing the correlation between serum homocysteine and serum ferritin levels.

A statistically significant negative correlation was observed between the serum vitamin B12 concentration and the serum homocysteine concentration (r=-0.285, p=0.004) (Table 2, Figure 1). A statistically non-significant positive correlation was noted between the serum folic acid and the serum homocysteine levels (r=0.033, p=0.748) (Figure 2) and between the serum ferritin and the serum homocysteine levels (r=0.179, p=0.075) (Figure 3).

**Table 2:** Correlation between serum homocysteine levels and other biochemical parameters. ^*^Significant.

Biochemical parameters		Serum vitamin B12	Serum folic acid	Serum ferritin
Serum homocysteine	Pearson correlation	-0.285	0.033	0.179
	P value	0.004*	0.748	0.075

The majority of the children (54%) were on monotherapy chelation (51 (51%) on deferasirox (DFX) monotherapy, three (3%) on deferiprone monotherapy), 36%(n=36) were on combination therapy (DFX and deferiprone), and 10% (n=10) were not on any chelation therapy. In children who were receiving monotherapy, the mean doses of DFX and deferiprone were 33.92±9.28 mg/kg and 56.66±20.13 mg/kg, respectively. In children receiving combination therapy, the mean doses of DFX and deferiprone were 34.44±8.76 mg/kg and 63.75±17.29 mg/kg, respectively. The mean duration of chelation therapy was 5.61±4.00 years. The mean serum ferritin concentrations were 2070.39±1213.16 ng/mL, 2613.33±1880.03 ng/mL, and 2644.42±1414.74 ng/mL for children on DFX monotherapy, deferiprone monotherapy, and combination therapy (DFX and deferiprone), respectively.

Maximum thalassemic children had macro-ovalocytosis (50%, n=50), followed by hypersegmented neutrophils in peripheral blood smears (31%, n=31). In these children, bald tongue was the most common clinical feature (50%, n=50), followed by glossitis/cheilitis/stomatitis (17%, n=17) and hyperpigmentation of the skin (15%, n=15). When children with macrocytosis and hypersegmented neutrophils were compared with children without macro-ovalocytosis and hypersegmented neutrophils, a statistically significant difference was observed in the mean serum values of homocysteine and vitamin B12. A statistically significant difference in the mean values of serum homocysteine was observed between children with and without glossitis/cheilitis/stomatitis. A statistically significant difference in the mean values of serum ferritin was observed between children with and without hyperpigmented skin (Table 3).

**Table 3:** Mean serum values and distribution based on clinical and laboratory characteristics. ^*^Significant.

Characteristics (N=100)		Frequency (%)	Serum homocysteine (µmol/L) Mean±SD	P value	Serum vitamin B12 (pg/mL) Mean±SD	P value	Serum folic acid (ng/mL) Mean±SD	P value	Serum ferritin (ng/mL) Mean±SD	P value
Macro-ovalocytosis	Present (n=50)	50	12.04±4.25	0.003*	121.52±39.27	0.001*	7.54±5.68	0.803	2384.86±1374.04	0.120
	Absent (n=50)	50	9.83±2.72		206.54±88.74		7.83±5.92		1966.70±1287.69	
Hypersegmented neutrophils	Present (n=31)	31	12.41±5.21	0.007*	108.45±38.55	0.002*	7.37±5.43	0.715	2458.77±1449.47	0.158
	Absent (n=69)	69	10.27±2.62		189.00±82.14		7.83±5.95		2048.64±1280.54	
Glossitis/cheilitis/ stomatitis	Present (n=17)	17	12.96±5.43	0.013*	157.59±53.20	0.719	7.64±5.24	0.970	2735.94±1561.88	0.058
	Absent (n=83)	83	10.52±3.15		165.35±85.25		7.70±5.91		2061.05±1272.00	
Hyperpigmentation of skin	Present (n=15)	15	11.99±4.64	0.237	144.67±59.58	0.315	6.63±3.16	0.447	2815.67±2070.55	0.045*
	Absent (n=85)	85	10.75±3.53		167.45±83.51		7.87±6.12		2062.86±1148.95	

## Discussion

Thalassemia syndromes represent the most common hemoglobinopathies worldwide. The goals of transfusion therapy are correction of anemia, suppression of erythropoiesis, and inhibition of gastrointestinal iron absorption. Nutritional deficiencies in vitamin B12 and folate, which occur due to ineffective erythropoiesis, can lead to varying degrees of disturbed homocysteine metabolism.

The recommended treatment for beta-thalassemia major involves regular red blood cell transfusions throughout life, which are usually administered every two weeks to maintain the pre-transfusion level of hemoglobin between 9 and 10.5 g/dL [1, 2]. In this study, the mean pre-transfusion hemoglobin level in the last six months was 8.23±1.02 g/dL, and the mean average number of transfusions in a year was 25.88±5.25, both of which indicate poor management of blood transfusion. In the studies by Sherief et al. and Mustafa et al., the mean pre-transfusion hemoglobin levels were 7.39±1.9 gm/dL and 8.78±0.86 gm/dL, respectively [7,8]. These results are in accordance with the results of the current study. In the current study, the mean age at the time of diagnosis of thalassemia was 9.22±3.71 months. Similar to the result of the current study, Grusel et al. [9] reported a mean age of 8.64 months at the time of diagnosis.

In our study, the majority (89%) of the children had normal serum homocysteine levels in the range of 5-15 µmol/l, while the mean serum homocysteine level was 10.93±3.72 µmol/L; 92% of the children had low serum vitamin B12 levels (less than 279 pg/mL), and the mean serum vitamin B12 level was 164.03±80.54 pg/mL. Fifty-three percent of the children had normal serum folic acid levels, with a range of 5-15 ng/mL and a mean value of 7.69±5.77 ng/mL. Ninety-seven percent of the children had high serum ferritin levels, whereas 3% of the children who were newly diagnosed had normal serum ferritin levels. In a previous study conducted by Sherief et al., the mean serum level of homocysteine was significantly higher in thalassemic children (9.57±0.23 µmol/L) than in the control group (8.61±0.49 µmol/L) [7]. The mean serum vitamin B12 level was significantly lower in thalassemic children (33.3±40.7 pg/mL) than in normal controls (332.7±136.4 pg/mL, p=0.001). The mean serum folic acid level was higher in thalassemic children (30.3±12.3 ng/mL) as compared to control subjects (17.7±2.4 ng/mL).

In this study, a statistically significant negative correlation was detected between the serum vitamin B12 concentration and the serum homocysteine concentration (r=-0.285, p=0.004). A positive correlation was detected between the serum folic acid concentration and the serum homocysteine concentration; however, this correlation was not statistically significant (r=0.033, p=0.748). A positive correlation was also detected between the serum ferritin and the serum homocysteine levels, but this correlation was not statistically significant (r=0.179, p=0.075). In a study by Mustafa et al., vitamin B12 was negatively correlated with homocysteine levels (r=-0.17, p=0.090), but this correlation was not statistically significant [8]. In a study conducted by Ozdem et al., the plasma homocysteine concentration did not correlate with either vitamin B12 (r=-0.26) or folic acid (r=-0.14) in thalassemic patients, which is contrary to our results [6]. In a study by Ghorban et al., a negative correlation was found between the serum ferritin and homocysteine levels (r=-0.26, p=0.451), but this correlation was not statistically significant [10].

In this study, the mean doses of DFX and deferiprone for children receiving monotherapy were 33.92±9.28 mg/kg and 56.66±20.13 mg/kg, respectively. For children receiving combination therapy, the mean doses of DFX and deferiprone were 34.44±8.76 mg/kg and 63.75±17.29 mg/kg, respectively. In the study by Totadri et al., the mean doses of DFX and deferiprone in combination therapy were 33.4±5.2 mg/kg (range: 20-40) and 84.8±8.5 mg/kg (range: 61-100), respectively [11]. In our study, the mean dose of deferiprone in children on combination therapy was lower than that in the above studies because many of these children avoided high doses of deferiprone pills due to side effects such as arthralgia. The mean serum ferritin concentrations in this study were 2070.39±1213.16 ng/mL, 2613.33±1880.03 ng/mL, and 2644.42±1414.74 ng/mL for children receiving DFX monotherapy, deferiprone monotherapy, and combination therapy, respectively (DFX and deferiprone). In the study by Gomber et al., the mean serum ferritin values were 3859.2 ng/mL, 3140 ng/mL, and 3696.5 ng/mL for children on DFX monotherapy, deferiprone monotherapy, and combination therapy (DFX and deferiprone), respectively, which were slightly higher than our values [12]. This difference can be explained by the greater mean age of the participants in their study, which ultimately would have led to an increase in ferritin if chelation therapy was not given adequately.

In this study, 50% of the children had macro-ovalocytosis, 31% had hypersegmented neutrophils, 10% had leukopenia, and 19% had thrombocytopenia in peripheral blood smears. In the study by Savage et al., 55.7% and 54.9% of children had macrocytosis, 16.9% and 17.9% of children had leukopenia, and 35.4% and 66.1% of children had thrombocytopenia due to cobalamin and folate deficiency, respectively [13]. In this study, 50% of the children had bald tongues, 17% had glossitis/cheilitis/stomatitis, and 15% had hyperpigmentation of the skin. In the study by Wang et al., atrophic glossitis was observed in 32.3% children, which is slightly higher compared to the current study [14]. We evaluated the neurological manifestations of vitamin B12 and folate deficiency, such as paresthesia, gait ataxia, peripheral neuropathy, and hand tremors, but in our study group, all these manifestations were found to be absent.

Limitations of the study

Serum homocysteine is a surrogate marker for detection of either vitamin B12 or folate deficiency, while methylmalonic acid is a surrogate marker for detection of vitamin B12 deficiency. Due to financial constraints and the non-availability of serum methylmalonic acid level, we could not establish whether the altered levels of homocysteine were due to folate deficiency or vitamin B12 deficiency. Similarly, the serum folate levels do not reflect the long-term folic acid status, and they are also altered with the recent oral folic acid intake. Red blood cell folate (RCF) levels reflect the exact long-term folic acid status. Due to financial issues, we could not investigate the RCF level, which might have provided more insight into folate status in these thalassemic children.

## Conclusions

Thalassemic children need robust management in the form of frequent blood transfusions and chelation therapy. The results of this study indicate that these thalassemic children are poorly managed, as the mean pre-transfusion hemoglobin level was below the recommended cutoff. Similarly, the management of iron overload with chelation therapy was neither appropriate nor the dose of chelation therapy was as per recommendation. As the majority of thalassemic children are deficient in vitamin B12, it may be prudent to screen all transfusion-dependent thalassemic children for vitamin B12 deficiency. Further studies to compare the vitamin B12 status of the general population with that of thalassemic children are needed to formulate guidelines regarding the routine supplementation of vitamin B12 in thalassemic children. Both macro- and hypersegmented neutrophils were significantly associated with high homocysteine levels and low vitamin B12 levels in children. Therefore, in a resource-limited setting, these laboratory features may be used as surrogate markers of low vitamin B12 and high serum homocysteine levels.
